# Rural family physician use of point-of-care ultrasonography: experiences of primary care providers in British Columbia, Canada

**DOI:** 10.1186/s12875-023-02128-z

**Published:** 2023-09-08

**Authors:** Jude Kornelsen, Hilary Ho, Virginia Robinson, Oron Frenkel

**Affiliations:** 1 Centre for Rural Health Research, Department of Family Practice, University of British Columbia, 3rd Floor David Strangway Building, 5950 University Boulevard, Vancouver, BC V6T 1Z3 Canada; 2 Rural Coordination Centre of British Columbia, 1665 West Broadway, Vancouver, BC V6J 1X1 Canada; 3 Providence Health Care, St. Paul’s Hospital, 1081 Burrard St, Vancouver, BC V6Z 1Y6 Canada

**Keywords:** Point-of-Care Ultrasound, Rural Medicine, Telemedicine, Virtual Medicine, Rural Health

## Abstract

**Background:**

In British Columbia (BC), rural and remote areas lack proximal access to radiographic services. Poor access to radiographic services in rural settings presents a challenge to timely diagnosis and screening across many disease states and healthy pregnancies. As a solution to the lack of access to radiographic services in rural settings, the Rural Coordination Centre of BC (RCCbc) supported rural Family Physicians (FPs) wishing to use PoCUS through the Intelligent Network for PoCUS (IN PoCUS) program. This study evaluates FPs’ experience and use of PoCUS in their clinical practice.

**Methods:**

This qualitative study conducted in-depth virtual interviews with 21 FPs across rural BC. The interview asked participants’ motivation to participate in the RCCbc program, the type of training they received, their current use of PoCUS, their experience with the technology, and their experience interacting with specialists in regional centres. Thematic analysis of findings was undertaken.

**Results:**

This study used Rogers’ framework on the five elements of diffusion of innovation to understand the factors that impede and enable the adoption of PoCUS in rural practice. Rural FPs in this study differentiated PoCUS from formal imaging done by specialists. The adoption of PoCUS was viewed as an extension of physical exams and was compatible with their values of providing generalist care. This study found that the use of PoCUS provided additional information that led to better clinical decision-making for triage and allowed FPs to determine the urgency for patient referral and transport to tertiary hospitals. FPs also reported an increase in job satisfaction with PoCUS use. Some barriers to using PoCUS included the time needed to be acquainted with the technology and learning how to integrate it into their clinical flow in a seamless manner.

**Conclusion:**

This study has demonstrated the importance of PoCUS in improving patient care and facilitating timely diagnosis and treatment. As the use of PoCUS among FPs is relatively new in Canada, larger infrastructure support such as improving billing structures, long-term subsidies, educational opportunities, and a quality improvement framework is needed to support the use of PoCUS among rural FPs.

**Supplementary Information:**

The online version contains supplementary material available at 10.1186/s12875-023-02128-z.

## Introduction

Increasingly, ultrasonography is used as part of clinical examinations among many specialities, including general medicine [1]. As a portable handheld device, Point-of-Care Ultrasonography (PoCUS) provides real-time feedback and is a valuable tool that can assist health care providers with diagnosis and procedural guidance [2]. Clinicians can use PoCUS as a bedside test to provide timely and accurate diagnoses [3, 4] or as an ancillary strategy as part of a comprehensive clinical assessment to facilitate the most appropriate course of care [5, 6]. For example, PoCUS has been shown to assist physicians when making decisions during resuscitation of patients with cardio-respiratory arrest by providing important information about the cause of the arrest and the quality of chest compressions [7].

While PoCUS is most often used in hospital settings [2], it has also been adopted by during critical patient transports by air and land, most notably for cardiac arrest and trauma. The diagnostic accuracy of in-flight ultrasound was comparable to those of imaging modalities used at the receiving hospitals, based on evidence from Canada and other countries [2].

A systematic review of PoCUS in rural and remote areas of Australia and other countries concurs with these findings. Studies show that when PoCUS was compared with clinical assessment, standard ultrasound or other imaging modalities, the accuracy was between 70 and 95%, depending on the patient’s condition [8]. The authors concluded that PoCUS improves patient care and is an essential tool in low resource settings. Barriers to the applications of PoCUS were noted, including lack of access to training, equipment and quality control, and difficulty maintaining competencies [8].

A systematic review of 27 studies showed significant variations in training for use of PoCUS during pregnancy, with training programs ranging in duration between three hours and two years. Multidisciplinary training programs were described in 44% of studies. Follow up training and skills assessment were not included in more than half of the PoCUS training programs published in the literature [9]. Preliminary studies of the use of PoCUS with COVID patients are promising and show that PoCUS is easy to use and decontaminate and can aid in the identification of peripheral changes in the lungs and the severity and progression of COVID-19 [10].

Rural communities across Canada face health service delivery challenges. There are gaps in local health care infrastructure, including access to diagnostic imaging services. In a survey of Canadian rural Emergency Departments (EDs), only 20% have a CT scanner and 28% have formal ultrasound services. To access advanced imaging, 44% of rural EDs have to transfer their patients to receive access to appropriate diagnosis services at major hospitals [11]. Consequently, rural patients often experience delays in receiving timely diagnosis and treatment [12]. A 2005 study by Lyon et al. demonstrated the benefits of PoCUS on rural ED decision making. The study showed that PoCUS reduced the number of differential diagnosis, altered patient management in 74% of cases and led to a more definitive diagnosis that avoided patient transfers in 53% of cases [13]. Studies have noted the efficacy of PoCUS in rural general medicine in improving clinical decisions and patient management in primary, in-patient, emergency, and obstetric care [14–17]. The utility and efficacy of PoCUS have been demonstrated in Canada and other countries, including Australia, New Zealand, Denmark, and the United States [18–20]. Moreover, the affordability and portability of PoCUS probes make access to ultrasonography accessible to rural FPs. The built-in WiFi, artificial intelligence-assisted diagnosis, and connectivity to smartphones and tablets render PoCUS a valuable tool for facilitating timely diagnoses and informing clinical decision-making with consultation from a specialist or peer [21].

Despite the many benefits of PoCUS, numerous studies continue to note limited uptake in PoCUS use. 72% of rural FPs in Whitehorse, Yukon Territory, stated that incorporating PoCUS in their practice would positively affect patient care and improve clinical decision-making. However, the lack of training opportunities, availability of probes, and personal confidence prevented FPs from fully incorporating PoCUS in their clinical practice [18]. Overall, PoCUS use in general practice remains limited despite the high degree of interest among rural FPs.

To increase ultrasound capacity and use in rural BC communities, British Columbia’s (BC) Joint Standing Committee on Rural Issues has provided funding through the Rural Coordination Centre of BC (RCCBc) to support rural Family Physicians’ who want to include PoCUS in their clinical practice. With this funding, 50 rural FPs across BC were issued ultrasound probes and training on the use of the probes. Physicians who received the probe were required to submit a one-page statement of interest and experience to the Rural Coordination Centre of BC. Those who met the criteria were chosen based on the time of their submission (those who submitted first were prioritized). As part of this “Intelligent Network for PoCUS” (IN PoCUS) program, recipients of the probes were asked to upload their ultrasound images onto an online platform to build a provincial reference database. Recipients had access to subsidized training programs, including the Hands-On Ultrasound Education OB course [22] and the Emergency Department Echo course [23], however, uptake was variable across the cohort. Participant education ranged from no additional education to taking a weekend course and doing mentorship training in a larger centre. In communities where colleagues had experience with PoCUS or specialists were available, either in person or through virtual consult through Rural Urgent Doctor in-aid (RUDi) [24], peer support was often noted as a valuable quality improvement mechanism. Phase I of this evaluation study explores how PoCUS is used in rural BC communities. Specifically, the objectives of the study were to:

1. Understand the experiences of participants in the IN PoCUS program (rural FPs using ultrasound probes);
2. Identify the scope of PoCUS practice and training for rural FP participants in the IN PoCUS program; and,
3. Understand the perspectives of key provincial and national stakeholders on rural PoCUS training and use.

## Methods

This qualitative study sought to understand the importance of and process through which PoCUS has been integrated into rural general practice in BC among a cohort of rural physicians who received subsidized Clarius probes from the Rural Coordination of BC (RCCBc). Given that generalist use of PoCUS, particularly in rural settings, is an emergent phenomenon, there has been a lack of in-depth qualitative research regarding care providers’ experiences of PoCUS. Qualitative data collection allows flexibility for participants to express their lived experience whereas quantitative research approaches such as surveys rely on questions with pre-defined response options. We adopted a qualitative framework for this study to ensure no assumptions regarding participants’ experiences were made.

### Participant selection

To be included in the study, participants had to be part of the IN PoCUS program. A letter of invitation was sent out to all 50 participants who were enrolled in the program. Out of 50 physicians who were invited, 21 physicians reached out to the research team to participate.

Participating physicians were offered a sessional rate, determined by the organization representing doctors in British Columbia.

### Interviews

Interviews were conducted over Zoom to minimize the exposure to and transmission of COVID-19. The interview guide covered topics on participants’ motivation to participate in the IN PoCUS program, the type of training they received, their current use of PoCUS, their experience with the technology, and their experience interacting with specialists in regional centres. The semi-structured interviews lasted between 22 and 67 minutes. Participants provided written and oral informed consent at the start of their interview to participate in the study and for their interview to be audio recorded for transcription purposes only. The interview questions can be found in Supplemental Material 1. All transcriptions and audio recordings were anonymized according to the regulations of University of British Columbia’s ethics board. This study was conducted according to the guidelines and regulations of University of British Columbia’s Behavioural Research Ethics Board (Ethics ID: H20-03356).

### Analysis

A thematic analysis framework outlined by Braun and Clarke (2006) was used to analyze the data [25]. One Research Assistant and two students analyzed the data independently using an inductive approach to develop salient themes and sub-themes from the interviews and compared findings to determine the degree of congruency. As there was a high level of agreement on the salient themes with only minor variations in semantics, a codebook was developed based on the themes articulated to guide the rest of the coding. This process led to a high degree of validity.

In the draft stages of the analysis, emergent findings were presented on several occasions to rural physicians and rural physician organizations for comments. We received feedback that there was a high level of congruence between our findings and the experiences of PoCUS users on the ground, corroborating our interpretation of the results and further confirming a high level of validity.

### Theoretical framework

We used Rogers’ (1962) theory on the Diffusion of Innovation (DOI) to understand FPs’ experiences of using PoCUS and the factors that led to the integration of PoCUS into their clinical practice [26]. Preliminary interviews with participants emphasized PoCUS as a rural practice innovation, and as such, we found it helpful to understand the data within the context of Roger’s theory. Although PoCUS technology itself is not new or innovative, its application in rural setting in British Columbia is. Though we did not plan the analysis based on Roger’s theory of innovation, and instead took an inductive approach to data analysis, the theory served as a valuable explanatory framework due to the congruence between the theory and our findings. DOI theory provides a model for understanding how an idea, product or process spreads through a population or social system over time, resulting in the adoption of a new practice. Rogers defined diffusion as “the process by which an innovation is communicated through certain channels over time among the members of a social system,” with successful diffusion contingent on the individual’s perception of the innovation [27](p5), [28](p 17–18). He noted the importance of exposure to the innovation across time as a key influence in adoption. Rogers developed a model that articulated a social process that includes different stages of adoption, each phase led by groups with distinct characteristics. There are five adopter categories: Innovators, Early Adopters, Early Majority, Late Majority, and Laggards. ‘Innovators’ are those who want to be the first to try the innovation (willing to take risks and in need of little encouragement). ‘Early Adopters’ are often opinion leaders who are comfortable adopting new ideas and only need implementation guidance (‘how-to’ guides). This group does not need to be coerced or convinced to adopt the innovation. IN PoCUS participants fall within Innovators and Early Adopters groups. The ‘Early Majority’ do not adopt new ideas before the average person and generally need to see evidence that the innovation works prior to adoption. The ‘late majority’ are more skeptical of change and will only adopt an innovation after it has been tried by the majority, whereas ‘Laggards’ are skeptical of and resistant to change and require pressure from others in the adopter groups to embrace the innovation.

The perception that the new practice is beneficial or improves existing practices is key to the adoption of innovation, and, as Rogers noted, it does not happen uniformly. Instead, it permeates through a population in discrete steps. Rogers (1962) also described five elements of an innovation or new technology that will determine the speed of its movement through the adoption phases [26]. They include:

1. Relative advantage - the “degree to which an innovation is perceived as better than the idea it supersedes”^26 (p15)^
2. Compatibility - the “degree to which an innovation is perceived as being consistent with the existing values, past experiences, and needs of potential adopters”^26 (p15)^
3. Complexity - the “degree to which an innovation is perceived as difficult to understand and use”^26 (p16)^
4. Trialability - the “degree to which an innovation may be experimented with on a limited basis”^26 (p16)^
5. Observability - the “degree to which the results of an innovation are visible to others”^26 (p16)^

**Fig. 1:**
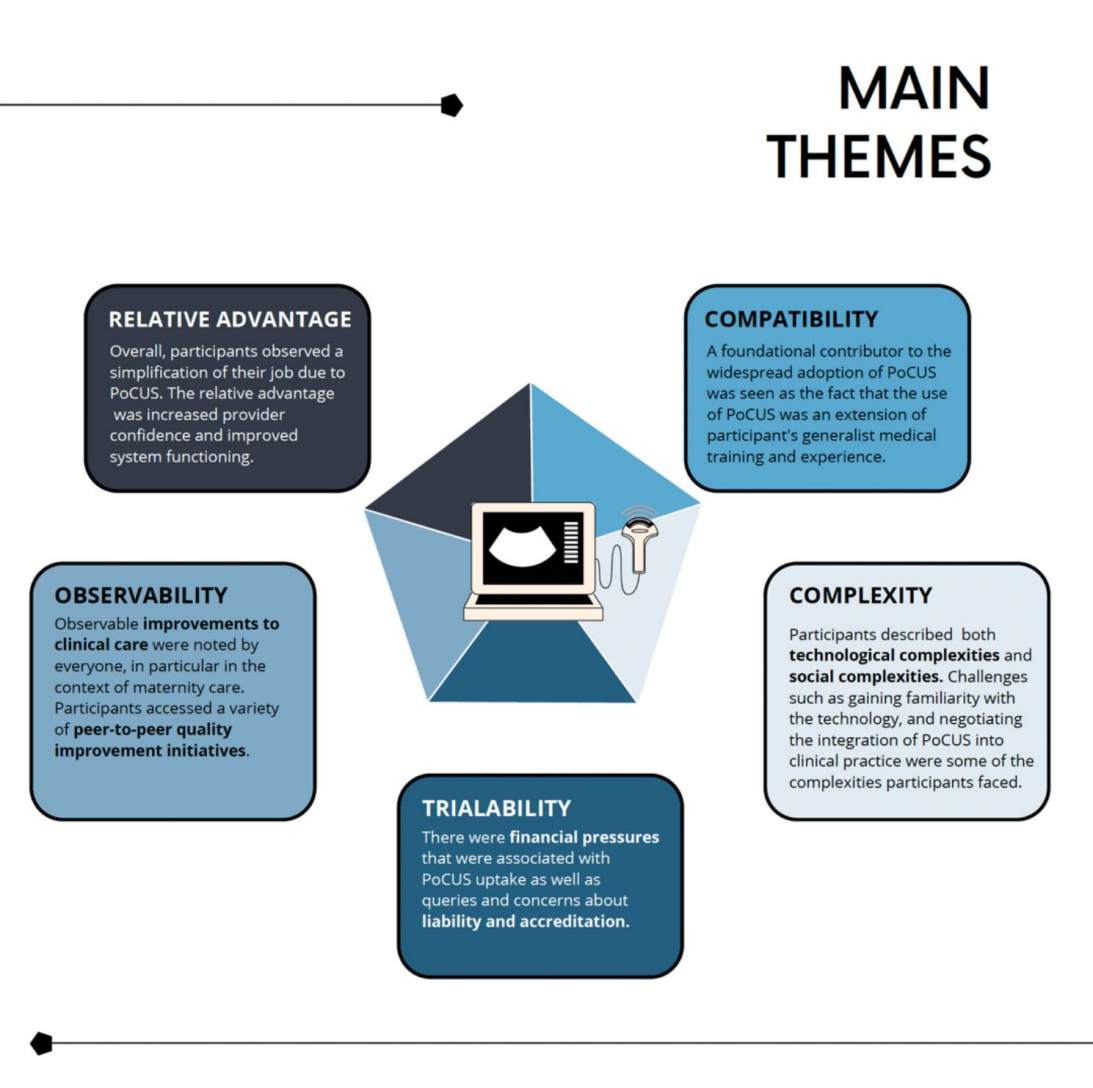
Visual summary of main themes

Findings from the interviews will be presented through these five characteristics. In this way, we can consider how social forces driving innovation led to the integration of PoCUS use in clinical practice and reveal the characteristics of ‘early adopters.’ See Fig. 1 for a visual summary of these themes.

## Results

### Relative advantage

Participants reported that the use of PoCUS provided a “relative advantage” in their clinical practice. PoCUS provided physicians with additional information that led to improved clinical decision making. The overarching value of PoCUS to rural practice was consistently expressed by participants with observations like, “It is a game-changer for small communities” and “[I]t’s an incredibly important tool that I don’t think I can practice in rural Canada without any longer.”

Many participants referenced the advantage of PoCUS to improve system functioning, specifically by reducing the draw on formal imaging services. Several participants emphasized the convergence of information gained from the scans with the patient’s history, physical exam, and other diagnostics led to better decision making and transfer avoidance. Participants noted the value of PoCUS in diagnosing fractures and heart failure. Additionally, PoCUS was an invaluable visual guide for procedures, such as inserting a central line or IUD. One participant noted the value of this specifically for maternity care:

I wasn’t sure [if] one of my… patients was breech. I didn’t have to send her to the hospital, I could just do a quick office ultrasound… or if they couldn’t find a fetal heart at 13 weeks when they should’ve been able to, they would send their patients to me instead of bothering the hospital. So, for obstetrical reasons, [I could] take the load off of the hospital.

Within the context of COVID-19, several participants noted the advantage of doing lung PoCUS. Some participants noted that although a COVID-19 diagnosis is based on the presentation of clinical symptoms, ultrasound was helpful as it provided additional clinical information. As one participant noted,

[It] might not change anything you’re going to necessarily do. However, it might change the conversation that you have with the patient. You can say, ‘From what I’m seeing, I now more strongly believe that you have a viral pneumonia such as COVID.’

Overall, participants in this study observed the simplification of their job due to PoCUS, and immediate feedback was extremely useful, particularly in communities without access to formal ultrasound services. As one participant noted,

But there are certain instances where you can get a positive [result], interpret it, make a clinical decision that changes the outcome and helps the patient a lot. So that’s where it really makes a difference for me.

Participants pointed out that the use of PoCUS increased their confidence and empowered them to provide better patient care, which led to increased job satisfaction. As one participant noted, “[I]t brings you back to the bedside. And so, it brings back the humanity of medicine for me in many ways.” Others noted how the availability of PoCUS facilitated recruitment to low volume sites, with interested candidates reassured of having colleagues who are early-adopters of PoCUS in the communities.

### Compatibility

Almost all participants in this study noted that the use of PoCUS was an extension of their generalist medical training and experience. Participants articulated their use of PoCUS as an extension of the physical exam or “extending the senses of a practitioner.” This sense of compatibility was seen as foundational to widespread adoption due to low barriers to usage. A participant expressed, “I don’t want to lose the quality of the ultrasound being an extension of the physical exam because that, to me, lowers the barrier to people using it.” Others expressed more directly their view of PoCUS as an extension of known skills. As one participant said:

I was teaching med students percussion… And I was like, this is just ultrasound, but old school… you’re basically using sound waves to try and detect fluid under a structure… And now, we have an ultrasound machine that can help us visualize what we were listening to before. [T]o me, that’s so powerful… We do need to make sure there’s safety, that it’s being used in a safe way.

For some, a sense of compatibility was reflected through the awareness of what their role vis-à-vis PoCUS was *not*: an equivalent or replacement of specialist diagnostic imaging. One physician pointed out, “A diagnostic scan is a very different kettle of fish with some very, very strict and clear parameters and is an incredibly useful tool. It’s just a different tool than point-of-care ultrasound.” Participants recognized specialized diagnostic imaging to be outside of their scope of practice, training, and comfort. For Family Physicians, the use of PoCUS aligned with their generalist education and training and informed their practice boundaries regarding what they were *not* comfortable doing. Participants had a strong sense of caution not to exceed their expertise, recognizing the potential for significant clinical consequences. As one participant observed:

I don’t have a problem saying ‘no, I’m not doing that. It’s not safe’… I can’t have that become the precedent or the kind of standard because it’s not the standard of care… [L]ike the DVT is a classic example: we’re trying to do the patient a favour, we’re trying to save them from having to travel, but if you miss an actual DVT and they have a PE and die, you haven’t done the patient a favor.

For most participants in this study, there was an overall appreciation of PoCUS as a clinical tool that is used to answer specific “yes-or-no” questions rather than a diagnostic test. When an unexpected finding did arise, participants noted the importance of a radiological consult. This was congruent with others who noted that a key attribute to rural PoCUS use was “being honest about your limitations.”

### Complexity

Participants in this study identified two levels of complexity in using PoCUS: technological complexity and a sense of social complexity arising out of the practice setting. The former was seen to be easily addressed with additional exposure to the technology, while solutions to the latter were less determined.

#### A. Technology complexity: familiarization period

Physicians expected a learning curve in getting acquainted with the new technology. Participants noted challenges with connectivity, including choice of connecting modality (Wifi, LAN, data). Other entry-level challenges included determining how to best physically incorporate the probe into the practice setting (“…do people carry it, do you put it in your pocket? How do you bring it to the bedside?”) and integrate it into their daily workflow, such as forgetting to charge the battery (of both the probe and the cell phone through which it works), and that the Clarius probe itself “can be finicky.” In the context of COVID-19, others noted difficulty negotiating the use of the probe in a bag to ensure a sterile environment and navigating the cleaning protocol to mitigate COVID-19 transmission. Regardless of the simplicity of addressing the perceived technological challenges, most participants noted that they were less inclined to use the technology when confronted with any technological perturbations.

#### B. Social complexity: challenges of ensuring accurate clinical diagnosis

Although most participants in this study reported that PoCUS was straightforward to adopt and use with minimal instruction, they expressed practice setting complexities of ensuring accurate clinical diagnosis. Many participants mentioned that they had to constantly navigate the boundaries of their training, experience, and the subsequent need to maintain “that index of clinical suspicion.” Relatedly, participants stressed the importance of recognizing when a scan would not be helpful or “knowing when to give up.” Several participants cautioned against “fishing expeditions” due to the danger of “finding something you are going to misinterpret.” Many participants referred to the value of “healthy fear” or, as one participant noted, the importance of having “respect for ultrasound before you start using it in your practice.” These social determinates of practice were imbued with social complexity and some participants recognized the propensity for rural health care providers to “go above and beyond.” Participants noted the tendency when working in low-resourced environments to “get pressed into spreading ourselves thinner and thinner, working miracles with nothing” while realizing as well that adverse events “end up on our shoulders.” Negotiating this tension came through as complexity of professional practice with regards to PoCUS.

#### C. Social complexity: negotiating the use of PoCUS scans

Participants expressed the social complexity of negotiating the function and use of PoCUS scans, which is traditionally a specialist domain. Formal scans are done upon the patients’ arrival to a larger centre, regardless of the conclusively of the PoCUS scans done, to confirm the diagnosis and assess for progression. In some instances, formal imaging was deemed unnecessary based on the availability of the bedside scans. Formal scans were forgone in instances when there was an existing and trusting relationship between rural physicians and specialists: “I sent a referral to the surgeon and said you know I’ve arranged formal imaging, but she, the surgeon, also knew that I had also done the fellowship and so she kind of took my word for it.” Although most agreed they would not make a specialist referral without an official scan, several mentioned they would include the results of a PoCUS to the radiologist as a rationale for an urgent scan.

Relatedly, most respondents reported minimal “pushback” from specialists and received helpful support for PoCUS in rural settings. As one noted, “I’d say that the vast majority of specialists that have been consulted where ultrasound is part of the clinical picture have been excited that we’re doing bedside ultrasounds.” Others noted specialists’ understanding of rural, low-resource practice settings and an appreciation that local providers do “whatever [they] can.” Although most respondents had positive consultation experiences, many also noted hearing otherwise from colleagues: “I’m also aware of situations where it hasn’t been as positive.” The minority of respondents who experienced a lack of support from regional specialists noted that the lack of support seemed to be due to the protocol of only reading images generated by Ultrasound Technicians. This created a sense of resignation for these providers.

### Trialability

In the context of PoCUS, “trialability” addresses the introduction into rural practice and the organic emergence of practice patterns, protocols, and the capacity to “course correct” based on observable process outcomes. As PoCUS is a relatively new protocol for generalist care providers, participants appreciated the emergence of practice patterns and conventions, developed in an iterative way that responds to the realities of rural practice. Participants noted the need to seek clarity and/or solutions in the following areas: integrating PoCUS into regular workflow patterns, financial considerations for using PoCUS (billing codes for generalist PoCUS use and subsidizing providers for technology and education), and creating clarity around the legal implications of PoCUS scans including regulatory guidance and accreditation.

#### A. Financial pressures associated with PoCUS uptake

Participants diverged on the ease with which they incorporated PoCUS into the context of their clinical practice based on whether they practiced in a fee-for-service or alternative payment setting. PoCUS learners expressed challenges with needing more time at the start to learn PoCUS (“[W]hen you’re still learning, it can slow you down. When you’re really adept, then it’s like you’re pulling it out on every patient because it’s what you’re using instead of a stethoscope, basically”). Consistently, those in an Alternative Payment Program (APP), or those in a salaried position, reported ease in integrating PoCUS into their practice. Conversely, those in a fee-for-service setting found the additional time required to learn and use PoCUS was incompatible with the efficiency of their practice. Additionally, a participant noted:

[T]here’s definitely people that I’m seeing in a family practice context where I’m not telling them I have an ultrasound… because it’s going to double the length of the appointment, and I can’t do that when I have a full waiting room. … It just makes my life so much more stressful when I have a whole bunch of people that now you’re getting further and further behind, and it’s so uncomfortable to be working on that situation.

Several participants in a fee-for-service environment noted a perceived advantage of creating billing codes for PoCUS scans to incentivize practice (such as in Ontario and Quebec): “...if you want something to get done, put a billing code on it, it’ll start getting done.”

Participants noted the value of funding for the technology itself, and many were wary of the financial barriers involved in keeping current with PoCUS. Many participants noted that they would not have a probe if the subsidy for the probes and training from the Rural Coordination Centre of BC were unavailable. Participants saw value to training programs beyond scanning and reading images to including “the ability to not be overconfident.” Some noted the amount of personal expenditure that would be incurred to improve efficiency and patient care: “[Y]ou provide better patient care but you won’t get any more remuneration, you’ll actually take a bit of a pay [cut] to pay it off and then the training and stuff to do with it…” Others noted the technological imperative towards improvement and obsolescence which can be challenging due to financial barriers of keeping up-to-date with the technology.

#### B. Liability and accreditation

Several study participants queried their legal liability for scans that led to a course of care, particularly when the objective of the scan may be to seek information to reduce unnecessary transfers out of the community. One participant asked specifically “[D]oes this suffice in place of a formal ultrasound? Am I putting myself at legal liability by doing a AAA screen and saying, ‘Well, they’re negative?’” Participants also noted a lack of guidance from professional bodies and a perception of the aversion to address the issue:

The college has no policy on ultrasound. And they need one, they need to decide what’s in line - what’s the scope of practice and what isn’t. And they may be forced to do that very shortly here.

Another participant noted “The college is going to have to issue a statement on their thoughts on point-of-care ultrasound replacing other modalities.”

Several participants raised the issue of accreditation within the emerging practice environment. Participants in this study expressed a desire for PoCUS training to be normalized and accessible by accrediting PoCUS. Participants often express “[PoCUS] should be part of and embedded into the training of anybody who’s doing bedside care.” However, participants were concerned about accrediting PoCUS, which could lead to additional barriers to using PoCUS:

Well, I actually hope it doesn’t get more regulated, to tell you the truth. I hope it doesn’t become [accredited]. I think… it is accessible to everyone [now]. We were trained to use it and know what we’re doing. And if we don’t know what we’re doing, we ask for help.

Further arguments were made against accreditation due to the value of real-time and real-world PoCUS training over courses “using standardized patients with no pathology,” as well as the pragmatic difficulty posed to rural providers if standardized Continuing Medical Education occurred outside of the community, requiring time away from practice. Underlying most arguments against accreditation was the sentiment that most physicians know their limitations and know when they need help and relying on this knowledge keeps the onus of responsibility on the individuals as opposed to with the system. Not all participants argued against accreditation, with some seeing the value to standardized Continuous Quality Improvement and the potential advantage of increased acceptance by specialists.

### Observability

#### A. Improvement to clinical care

Observable results of rural PoCUS to clinical practice were noted by everyone who participated in the study. Most interview participants shared anecdotes of positive responses from patients due to the immediate information provided on their clinical condition. This was particularly observed with maternity patients who could be easily reassured about the viability of a pregnancy or the in-utero position of a baby at term. In all instances, the capacity of a simple scan to avoid referral out of the community for formal imaging was appreciated.

Participants who participated in this study described PoCUS as a “game changer”, “essential to rural practice”, “I could never go back”, “amazing potential” and “better decision-making.” As one participant described, they are those who had “drunk the Kool-Aide.” The two quotes below capture the transformative impact PoCUS has on rural health care:

[I]t’s hard to know where to start. [T]here’s very rarely a day goes by that it isn’t helping with care in a substantial way.

I just want to leave you guys with the impression that point-of-care ultrasound is a game-changer for these small communities.

#### B. Peer-to-peer quality improvement initiatives

The final layer of “observability” for participants in this study was gained through mentorship and Quality Improvement (QI) initiatives, with the caveat that all participants noted the lack of such formal programs. This *lack* of observability in their rural PoCUS practice was noted, namely the idea of “you don’t know what you don’t know because you are always working solo.” Almost all participants voiced the value of mentorship and formal review of scans, suggesting that isolated work made improvement difficult.

To this end, participants developed informal networks and peer support systems to assess the quality of their work and created processes such as parallel studies. One participant explained, “I’m the only one in most the places where I work who’s comfortable making a diagnosis or ruling out a diagnosis using lung ultrasound. So, what I do is I often order a chest x-ray in parallel.” Others relied on “scanning and scanning again” while most took advantage of peer review by other physicians in their community. As one participant noted, “...we’re always helping each other out. Someone can call me from the clinic and say, ‘Hey, could you help me with the scan?’ or ‘What do you think of this?’” Others took a more formal approach of accessing funding for local training and dedicated teaching time. Some participants noted the strategy for reaching a “critical mass” of PoCUS users in their community to be a stable resource for others who want to develop or maintain their skills.

A smaller group of respondents recalled accessing provincial resources for support, including the Rural Urgent Doctors In-aide (RUDI), a virtual practice support line staffed by physicians who offered guidance with PoCUS. Some noted opportunities through UBC’s Coaching and Mentoring Program (CAMP) for one-on-one support. Others accessed supportive provincial experts for feedback on scans, although in an ad-hoc way. These additional resources allow rural physicians to develop their skills further and further demonstrate the value and utility of PoCUS in their clinical practice.

### Limitations

Rural Family Physicians who received subsidized Clarius probes from RCCbc were highly motivated to take up PoCUS and went through a low-barrier screening process to obtain the probe that assessed enthusiasm and commitment more than experience and training. Approximately half of this group of highly motivated providers participated in the interview. Participants in the study volunteered their time to receive and learn to use the subsidized probes and took the time to contribute this study. Thus, participants in the study were naturally predisposed to being rural PoCUS champions. Although this study cohort may not represent the entire population of rural PoCUS users, the consistency of experience and value attribution of PoCUS by participants suggests a shared experience that is likely extrapolatable to the larger population.

## Discussion and conclusion

Results from this study revealed the value and efficacy of PoCUS from the perspective of rural, generalist health care providers. The importance of care providers’ job satisfaction in health care sustainability has been well-recognized and documented and is now included in the Institute for Healthcare Improvement’s Quaduple Aim (along with better health outcomes, better patient satisfaction and lower costs) [29, 30]. Qualitative evidence attested to increased job satisfaction and improved clinical decision-making from the adoption of PoCUS, both of which are lightning rods to optimizing patient care and outcomes. While PoCUS enables rural FPs to make better clinical decisions and thereby improve patient management, it is crucial to recognize that the use of PoCUS is not a surrogate for specialist imaging services. Instead, PoCUS functions as an additional resource for rural FPs in low-resource settings. To this end, there is clear indication for the appropriate scope of practice for generalist, point-of-care ultrasound. A systematic review of 51 studies describing the use of PoCUS in general practise found that family physicians most often used PoCUS for abdominal and obstetric/gynecological scans [31]. Hours of training ranged from 2 to 320 hours, although research shows that the quality of the ultrasonography depends less on training hours and more on the type of examination. False positive findings were much higher for cardiac examinations performed by general practitioners than obstetric or abdominal scans. The authors conclude that the risk of harm is lowest when family physicians use PoCUS to verify a diagnosis [31]. A study of rural general practitioners who used PoCUS in New Zealand revealed the need to agree on scope and standards to increase the benefits and safety of rural PoCUs use [32].

It is essential to understand the contextual realities of rural practice when evaluating the use of PoCUS. Lower procedural volume reflected by low population densities and the consequent reduced availability of local specialists and specialized imaging services can lead to a high volume of transfers out of the community. For example, uncertainty around the position of a fetus at term is a concern for those in rural communities that may not have the capacity to support breech deliveries. Strategies to reduce unnecessary transfers – such as the availability of PoCUS to visualize fetal position or rule out worrisome conditions – are an essential component of stabilizing rural health services and supporting rural health care providers. In one prospective study of 574 patients of general practitioners in Denmark, use of PoCus was linked to increased confidence in the diagnosis for 89% of patients, a change in treatment plan for 27% of patients, and an absolute reduction in the need for referral to secondary care from 49 to 26% [33].

From a systems perspective, scaling up the use of PoCUS in rural areas necessitates system-level subsidies to minimize the costs of both technology and education. As several participants in this study noted, the increased health systems efficiency gained through PoCUS is not reflected through reduced technology costs or increased potential for billing. In addition, Quality Improvement mechanisms for PoCUS users should include real-time case reviews by the community of rural PoCUS users to ensure the maintenance of safety and quality. The results of this process should be made transparent and available to the wider community through aggregate reporting that respects the privacy of individual providers and the confidentiality of patients.

Based on primary data from this study, recommendations to support rural generalist PoCUS use should be consolidated around user support by promoting program-level facilitators. These supports include skills development and skill maintenance to address low patient volume endemic in many rural communities with low population densities through ongoing training sessions, both with experienced users and in peer-to-peer settings. Closely aligned is the need for infrastructure and support for PoCUS mentors who can be reached at any time to provide immediate consultation on scans and facilitate further skill development. Specifically, the mentor may provide consultations on image generation, image interpretation, and one-to-one coaching and skill development. Cumulatively, these initiatives would lead to a PoCUS community of practice to facilitate learning and knowledge exchange and reduce the reality of rural isolation from colleagues. Due to the realities of provider locations across rural geographies, this could be facilitated virtually through online forums and other modalities of virtual communication. Finally, all initiatives must be undertaken within a formalized Continuous Quality Improvement framework, including developing mechanisms for engaging with peers and specialist colleagues to review scans and improve image generation.

International evidence on the use of PoCUS suggests both safety and efficacy amongst generalist users [13, 18–20]. Findings from this study in rural BC reflect the value of PoCUS in rural and isolated settings in improving patient care (mainly through reduced transfers out of the community for diagnosis and treatment) and increasing job satisfaction and retention by adding to the “toolbox” of isolated care providers. Integration of PoCUS into clinical practice has been facilitated in BC by perceptions of the technology’s relative advantage to current practice, compatibility with existing experiences and skills, ease of use, the potential for improvement in the care provided, and observable advantage of use. Ongoing system-level support will optimize the integration and use of PoCUS in rural settings, leading to improved patient outcomes. From this perspective, support for use is imperative for health system decision-makers.

### Electronic supplementary material

Below is the link to the electronic supplementary material.

Supplementary Material 1

## Data Availability

The anonymized transcriptions and analytic datasets are stored at the Centre for Rural Health Research at University of British Columbia, according to the university’s data policy. Anonymized data are available from the corresponding author on reasonable request.

## References

[1] Moore CLCJ (2011). Point-of-care ultrasonography. N Engl J Med.

[2] Shekhar AC, Blumen I (2021). A narrative review on the use of ultrasonography in critical care transport: is POCUS hocus?. Trends in Anaesthesia and Critical Care.

[3] Laursen CB, Sloth E, Lassen AT, Rd C, Lambrechtsen J, Madsen PH (2014). Point-of-care ultrasonography in patients admitted with respiratory symptoms: a single-blind, randomized controlled trial. Lancet Resp Med.

[4] Smallwood N, Dachsel M (2018). Point-of-care ultrasound (POCUS): unnecessary gadgetry or evidence-based medicine. Clin Med (Lond).

[5] Weile J, Brix J, Moellekaer AB (2018). Is point-of-care ultrasound disruptive innovation? Formulating why POCUS is different from conventional comprehensive ultrasound. Crit Ultrasound J.

[6] Competency Training Requirements for the Area of Focused Competence in Acute Care Point-of-Care Ultrasonography. 2019 Dec [cited 2021Mar]. Available from: https://www.royalcollege.ca/rcsite/documents/ibd/acute-care-pocus-ctr-e.pdf.

[7] Ávila-Reyes D, Acevedo-Cardona AO, Gómez-González JF, Echeverry-Piedrahita DR, Aguirre-Flórez M, Giraldo-Diaconeasa A (2021). Point-of-care ultrasound in cardiorespiratory arrest (POCUS-CA): narrative review article. Ultrasound J.

[8] Shaddock L, Smith T (2022). Potential for Use of Portable Ultrasound Devices in Rural and Remote Settings in Australia and other developed countries: a systematic review. J Multidisciplinary Healthc.

[9] Bidner A, Bezak E, Parange N (2022). Evaluation of antenatal point-of-care Ultrasound (PoCUS) training: a systematic review. Med Educ Online.

[10] Gandhi D, Jain N, Khanna K, Li S, Patel L, Gupta N. Current role of imaging in COVID-19 infection with recent recommendations of point of care ultrasound in the contagion: a narrative review. Annals of Translational Medicine. 2020;8(17). 10.21037/atm-20-3043 PMC7576001 33145313

[11] Fleet R, Poitras J, Maltais-Giguere J (2013). A descriptive study of access to services in a random sample of canadian rural emergency departments. BMJ Open.

[12] Sounness B, Hughes C, Winzenberg T (2008). Rural GPs’ satisfaction with radiology services to their communities: a qualitative study. Rural Remote Health.

[13] Lyon M, Blaivas M, Brannam L (2005). Use of emergency ultrasound in a rural ED with limited radiology services. Am J Emerg Med.

[14] Nicola R, Dogra V (2016). Ultrasound: the triage tool in the emergency department: using ultrasound first. Br J Radiol.

[15] Nixon G, Blattner K, Koroheke-Rogers M, Muirhead J, Finnie WL, Lawrenson R, Kerse N (2018). Point-of-care ultrasound in rural New Zealand: Safety, quality and impact on patient management. Aust J Rural Health.

[16] Léger P, Fleet R, Maltais-Giguère J, Plant J, Piette É, Légaré F, Poitras J (2015). A majority of rural emergency departments in the province of Quebec use point-of-care ultrasound: a cross-sectional survey. BMC Emerg Med.

[17] Becker DM, Tafoya CA, Becker SL, Kruger GH, Tafoya MJ, Becker TK (2016). The use of portable ultrasound devices in low- and middle-income countries: a systematic review of the literature. Trop Med Int Health.

[18] Siu T, Chau H, Myhre D. Bedside ultrasonography performed by family physicians in outpatient medical offices in Whitehorse, Yukon. Can J Rural Med. 2013 Spring;18(2):43–6. PMID: 23566861. 23566861

[19] Arnold AC, Fleet R, Lim D (2021). A case for mandatory ultrasound training for rural general practitioners: a commentary. Rural Remote Health.

[20] Andersen CA, Davidsen AS, Brodersen J, Graumann O, Jensen MB (2019). Danish general practitioners have found their own way of using point-of-care ultrasonography in primary care: a qualitative study. BMC Fam Pract.

[21] Britton N, Miller MA, Safadi S, Siegel A, Levine AR, McCurdy MT (2019). Tele-Ultrasound in Resource-Limited Settings: a systematic review. Front Public Health.

[22] Hands-On Ultrasound Education. Rural Coordination Centre of BC. [cited 2022Sept]. Available from: https://rccbc.ca/education-and-cmecpd/cmecpd/rural-courses/obstetrical-ultrasound/.

[23] Emergency Department Echo Training Course. [cited 2022Sept]. Available from: https://edecourse.com/.

[24] Rural Urgent Doctor in-aid Real Time Virtual Support. Rural Coordination Centre of BC. cited 2022Sept]. Available from: https://rccbc.ca/rtvs/rtvs-pathways/rudi/.

[25] Braun V, Clarke V. Using thematic analysis in psychology. Qualitative Res Psychol. 2006Jul;3(2):77–101.

[26] Rogers EM (1962). Diffusion of innovations.

[27] Rogers EM (1983). Diffusion of innovations Glencoe.

[28] Rogers EM (1995). Diffusion of innovations.

[29] Sibeoni J, Bellon-Champel L, Verneuil L, Siaugues C, Revah-Levy A, Farges O (2021). Workplace environment around physicians’ burnout: a qualitative study in french hospitals. Scand J Work Environ Health.

[30] Feeley D. The triple aim or the quadruple aim? Four points to help set your strategy [Internet]. Institute for Healthcare Improvement. 2017 [cited 2021Aug26]. Available from: http://www.ihi.org/communities/blogs/the-triple-aim-or-the-quadruple-aim-four-points-to-help-set-your-strategy.

[31] Andersen CA, Holden S, Vela J, Rathleff MS, Jensen MB (2019). Point-of-care Ultrasound in General Practice: a systematic review. Ann Fam Med.

[32] Nixon G, Blattner K, Muirhead J, Finnie W, Lawrenson R, Kerse N (2018). Scope of point-of-care ultrasound practice in rural New Zealand. J Prim Health Care.

[33] Andersen CA, Brodersen J, Davidsen AS, Graumann O, Jensen MB (2020). Use and impact of point-of-care ultrasonography in general practice: a prospective observational study. BMJ open.

