# Supplementary material for: Rural family physician use of point-of-care ultrasonography: experiences of primary care providers in British Columbia, Canada
Source: BMC Prim Care. 2023 Sep 8;24:183. doi: 10.1186/s12875-023-02128-z (PMC10486031; doi:10.1186/s12875-023-02128-z)
Supplement: Supplementary file 1 — Supplementary Material 1 [file 12875_2023_2128_MOESM1_ESM.docx]

Supplemental File 1: Interview questions

| **Interview questions for family physicians:** |
| --- |
| Level of training/confidence |
| 1. Please describe any training you received in PoCUS?  2. What specific organizations are providing you with training?  3. What would the ideal training scenario be for you?  4. How confident are you in your abilities in PoCUS?  5. Are there any patients/conditions for which you feel more/less confident to use PoCUS?  6. Do you feel like you have any gaps in your training?  7. Do you ever worry about negative implications from PoCUS regarding patient's  understanding of the technology’s accuracy |
| Current use of PoCUS |
| 1. How has PoCUS affected the overall clinical care you provide?  2. What patients/indications are you using PoCUS for? Have you expanded your use of  PoCUS since receiving your training?  3. How is PoCUS affecting your care and/or management of patients/conditions?  4. Can you give us any examples of how PoCUS has improved your practice?  5. Have you ever experienced that using PoCUS has taken time away from other important  clinical activities? |
| Experience interfacing with specialists at regional referral centers regarding use of PoCUS |
| 1. How much do you rely on regional referral specialists in your use of PoCUS?  2. Has your referral pattern shifted at all?  3. Do you feel regional specialists are supportive of rural FP PoCUS?  Interviews with the specialist cohort will focus on their views regarding FPs performing PoCUS. |
| **Interview questions for specialists:** |
| 1. Do you see a need for Family Physician (FP) PoCUS in rural settings?  2. Do you have any concerns with PoCUS in rural settings?  3. Are there certain conditions and/or patient populations where you believe FP PoCUS use  is more/less beneficial?  4. What are your thoughts/perceptions/attitudes regarding the use of PoCUS by FP? |
